# Activation of NRF2 and ATF4 Signaling by the Pro-Glutathione Molecule I-152, a Co-Drug of *N*-Acetyl-Cysteine and Cysteamine

**DOI:** 10.3390/antiox10020175

**Published:** 2021-01-26

**Authors:** Rita Crinelli, Carolina Zara, Luca Galluzzi, Gloria Buffi, Chiara Ceccarini, Michael Smietana, Michele Mari, Mauro Magnani, Alessandra Fraternale

**Affiliations:** 1 Department of Biomolecular Sciences, University of Urbino Carlo Bo, 61029 Urbino, Italy; c.zara@campus.uniurb.it (C.Z.); luca.galluzzi@uniurb.it (L.G.); g.buffi@campus.uniurb.it (G.B.); c.ceccarini2@campus.uniurb.it (C.C.); michele.mari@uniurb.it (M.M.); mauro.magnani@uniurb.it (M.M.); alessandra.fraternale@uniurb.it (A.F.); 2 Institut des Biomolécules Max Mousseron, Université de Montpellier UMR 5247 CNRS, ENSCM, 34095 Montpellier, France; michael.smietana@umontpellier.fr

**Keywords:** GSH, Cysteamine, N-acetyl cysteine, KEAP1, NRF2, ATF4

## Abstract

I-152 combines two pro-glutathione (GSH) molecules, namely N-acetyl-cysteine (NAC) and cysteamine (MEA), to improve their potency. The co-drug efficiently increases/replenishes GSH levels in vitro and in vivo; little is known about its mechanism of action. Here we demonstrate that I-152 not only supplies GSH precursors, but also activates the antioxidant kelch-like ECH-associated protein 1/nuclear factor E2-related factor 2 (KEAP1/NRF2) pathway. The mechanism involves disulfide bond formation between KEAP1 cysteine residues, NRF2 stabilization and enhanced expression of the γ-glutamil cysteine ligase regulatory subunit. Accordingly, a significant increase in GSH levels, not reproduced by treatment with NAC or MEA alone, was found. Compared to its parent compounds, I-152 delivered NAC more efficiently within cells and displayed increased reactivity to KEAP1 compared to MEA. While at all the concentrations tested, I-152 activated the NRF2 pathway; high doses caused co-activation of activating transcription factor 4 (ATF4) and ATF4-dependent gene expression through a mechanism involving Atf4 transcriptional activation rather than preferential mRNA translation. In this case, GSH levels tended to decrease over time, and a reduction in cell proliferation/survival was observed, highlighting that there is a concentration threshold which determines the transition from advantageous to adverse effects. This body of evidence provides a molecular framework for the pro-GSH activity and dose-dependent effects of I-152 and shows how synergism and cross reactivity between different thiol species could be exploited to develop more potent drugs.

## 1. Introduction

Reduced glutathione is the most abundant non-protein thiol specie within the cell, where it plays a crucial role in controlling redox homeostasis, metabolism, detoxification, and signal transduction [1]. Maintenance of adequate GSH levels is fundamental to proper cell functioning, proliferation and survival under physiological and stress conditions.

Glutathione is synthesized in a two-step process consisting of enzymatic reactions catalysed by ɣ-glutamyl-cysteine ligase (GCL, EC 6.3.2.2) and glutathione synthase (GS, EC 6.3.2.3). The rate of glutathione synthesis is essentially dependent on cysteine levels and GCL activity. GCL is composed of a catalytic (GCLC) and modifier (GCLM) subunit; the catalytic monomer functions autonomously, but its association with the regulatory subunit greatly increases the Km for glutamate and ATP and the Ki for GSH [2].

Notably, GCL activity is mainly regulated at the level of transcription, although other levels of regulation may occur post-translationally [3]. Both GCL subunits are under the transcriptional control of NRF2, which is activated by many oxidants and electrophilic compounds [4,5]. The classical mechanism of activation involves alkylation and/or oxidation of redox-sensitive cysteine residues on KEAP1, an E3 ligase adaptor protein, which, under basal conditions, targets NRF2 to proteasome-mediated degradation, keeping NRF2 levels low [6]. Upon oxidation/alkylation, KEAP1 becomes non-functional with the consequent stabilization and translocation of NRF2 to the nucleus, where the factor drives the transcription of a set of genes involved in glutathione synthesis and recycling, xenobiotic metabolism and transport, and antioxidant genes [7].

ATF4 is a cAMP-response element binding protein that belongs to the activating transcription factor/cAMP response element-binding protein family (ATF/CREB) [8]. ATF4 is known as the main effector of the integrated stress response (IRS), an adaptive pathway where different stress stimuli signal disturbance in cell homeostasis, converging on a common transducer, namely the eukaryotic translation initiation factor 2α (eIF2α). In this pathway, phosphorylation of eIF2α leads to global attenuation of Cap-dependent translation, while concomitantly initiating the preferential translation of specific mRNAs such as the ATF4 mRNA [9]. Under stressful conditions, elevated translation of ATF4 facilitates transcriptional upregulation of protective or pro-apoptotic stress-responsive genes, depending on the nature, intensity, and duration of the stress stimuli.

A growing body of evidence suggests that simultaneous activation of NRF2 and ATF4 potentiates the expression of cytoprotective genes and increases GSH levels under both basal and oxidative stress conditions [10,11]. Although the NRF2 and ATF4 signaling pathways have independent mechanisms of activation, they do indeed share some downstream target genes [12,13]. Moreover, NRF2 and ATF4 cooperate on the level of glutathione, where ATF4 promotes the uptake of glutathione amino acid building blocks, including glutamine and cysteine, and promotes glutamate production via induction of asparagine synthetase [14,15]. On the other hand, ATF4 is also involved in the degradation of GSH under stress conditions by inducing the expression of the cation transport regulator-like protein 1 (CHAC1; EC 4.3.2.7) [16]. CHAC1 belongs to a family of enzymes responsible for the degradation of intracellular glutathione in response to different types of stresses to provide three vital amino acids and help cells to cope with stress [17].

Disturbances in glutathione homeostasis have been implicated in the etiology and/or progression of several human diseases, including cancer, cardiovascular, and neurodegenerative disorders [18]. Attempts to replenish GSH levels in chronic and transient depletion states have focused on the use of GSH or GSH analogues and biosynthetic precursors such as L-cysteine. More recently, approaches related to the possibility of manipulating the activity of the enzymes involved in GSH homeostasis have also been taken into consideration [19,20]. These approaches involve the use of natural or chemically synthesized compounds able to activate antioxidant signalling pathways, although for most of these substances a clear mechanism of action has not been provided.

I-152 is a conjugate of NAC and S-acetyl-MEA (SMEA) linked together by an amide bond [21]. The molecule is deacetylated on the MEA moiety and hydrolyzed within the cells to release NAC and MEA, two well-known pro-glutathione compounds. I-152 has been shown to efficiently boost GSH both in vivo and in vitro, under physiological and pathological conditions characterized by GSH depletion (i.e., viral infection) (reviewed in [22]). Little is known about the mechanism of action of I-152. To date, its beneficial effects on GSH metabolism have been attributed to its ability to provide cysteine precursors.

In this investigation, we show that I-152 activates the NRF2 signaling pathway in RAW 264.7 cells by inducing KEAP-1 oxidation and NRF2 stabilization, leading to GCLM expression and increased intracellular GSH levels. The same effects could not be reproduced using equimolar concentrations of NAC or MEA. Notably, while I-152 dose-dependently activated NRF2 at all the doses tested, only high doses induced transcriptional activation of ATF4 and ATF4-dependent gene expression. Under this condition, GSH levels did not change, but rather tended to decrease over time. This paradoxical effect and the possible consequences of the concomitant activation of NRF2 and ATF4 are discussed.

## 2. Materials and Methods

### 2.1. Cell Culture and Treatment

The murine macrophage cell line RAW 264.7 was cultured in Dulbecco’s Modified Eagle’s Medium (DMEM) high glucose supplemented with 10% heat-inactivated fetal bovine serum, 2 mM L-glutamine, 100 μg/mL streptomycin and 100 U/mL penicillin. Cells were plated in 35 mm dishes at a density of 0.1 × 10^6^ cells/well two days before the treatment. I-152 was synthesized as previously described [21], while cysteamine and N-acetyl cysteine were purchased from Sigma-Aldrich. The chemicals were directly dissolved into the cell culture medium, which was then filtered through a sterile 0.22 µm pore size membrane. On the day of the treatment, the culture medium was removed and replaced with a fresh one containing or not containing the indicated molecules.

### 2.2. Cell Lysates

Cells were washed on ice with cold Phosphate-Buffered Saline (PBS) and directly lysed in Sodium Dodecyl Sulfate (SDS) buffer (50 mM Tris-HCl, pH 7.8, 0.25 M sucrose, 2% (*w/v*) SDS, 10 mM *N*-ethylmaleimide-NEM) supplemented with a cocktail of protease (Complete, Roche) and phosphatase inhibitors (1 mM Na_3_VO_4_, 1 mM NaF). Whole cell lysates were boiled for 5 min, sonicated at 70 Watts for 40 s to shear nucleic acids and centrifuged at 14,000× *g* at room temperature (RT) to remove debris. Protein content was determined in the supernatant by the Lowry Assay using bovine serum albumin (BSA) as a reference standard.

### 2.3. SDS-PAGE and Western Immunoblotting

Proteins were resolved by SDS polyacrylamide gel electrophoresis (SDS-PAGE) and electroblotted onto polyvinylidene difluoride (PVDF) membranes (0.2 µm pore size). After blocking in 5% (*w/v*) nonfat dry milk (Cell Signaling Technologies, #9999), membranes were incubated with the following primary antibodies: anti NRF2 (D1Z9C, XP #12721), anti KEAP1 (D1G10, #7705), anti p53 (1C12, #2524), anti ATF4 (D4B8, #11815), anti [P]eIF2α (Ser51) (D9G8, XP #3398) and anti β-ACTIN (#4967) from Cell Signaling Technologies; anti HMOX1 (F-4, sc-390991) and anti GADD153 (CHOP) (B-3, sc-7351) (from Santa Cruz Biotechnology; anti GCLC (VPA00695) from BioRad; anti GCLM (A5314) from ABclonal; anti CHAC1 (N1C3, GTX120775) from GeneTex. After overnight incubation at +4 °C, a horseradish peroxidase (HRP)-conjugated secondary antibody (Bio-Rad, Hercules, CA, USA) was used to detect immunoreactive bands. Bands were visualized with the enhanced chemiluminescence detection kit WesternBright ECL (Advansta, San Jose, CA, USA) in a ChemiDoc MP Imaging System and quantified by using the Image Lab software (Bio-Rad, Hercules, CA, USA).

### 2.4. Cycloheximide (CHX) Chase Assay and Relative Half-Life Determination

Cells were left untreated or treated with 1 mM I-152. CHX (50 µg/mL) was added to the culture medium of both untreated and I-152-treated cells at the beginning of the incubation (co-treatment) or after 30 min of pre-incubation with medium containing or not containing the I-152 molecule. Cells were collected immediately (time 0) and at specific time points (10, 20, 30, and 60 min) following CHX administration. Whole cell lysates were obtained as described above and separated by SDS-PAGE for Western blotting analysis. To determine the relative half-life, the intensity of the immunoreactive bands was measured and expressed as the percent of the time 0 sample value (initial value). The Log_10_ of the percentage values was plotted versus time (min). After linear regression analysis, the time required for degradation of 50% of the protein from its initial value (half-life) was calculated from the equation by replacing y with Log_10_ of 50 and solving for x.

### 2.5. NEM-Alkylated Redox Western Blotting

Cells were washed with cold PBS and scraped in 10% (*w/v*) trichloroacetic acid (TCA). After 30 min on ice, samples were centrifuged at 14,000× *g* + 4 °C, and the pellets were washed twice in ice-cold acetone. Protein pellets were dissolved by vortexing in 100 mM Tris-HCl, pH 6.8, 2% (*w/v*) SDS, 40 mM NEM. NEM was used to block free sulfhydryl groups and avoid Cys oxidation during extraction. Protein extracts were centrifuged again to remove insoluble debris, and protein concentration was determined by the Lowry Method. Protein samples were separated by non-reducing SDS-PAGE. Parallel runs were performed under reducing conditions to demonstrate the specificity of the signal.

### 2.6. RNA Isolation and cDNA Synthesis

RAW 264.7 cells were directly lysed with 700 μL of QIAzol^®^ Lysis Reagent (Qiagen, Hilden, Germany). Total RNA was isolated using the miRNeasy Mini Kit (Qiagen, Hilden, Germany) and eluted in 40 µL of RNase-free water. The extracted RNA was quantified using a NanoVue PlusTM spectrophotometer (GE Healthcare Life Sciences, Piscataway, NJ, USA). Total RNA (500 ng) was reverse transcribed using PrimeScriptTM RT Master Mix (Perfect Real Time) (Takara Bio Europe, Saint-Germain-en-Laye, France) according to the manufacturer’s instructions.

### 2.7. Quantitative Real-Time PCR

The expression of *Atf4*, *Chac1*, *Chop*, *Gclc*, *Gclm*, *Hmox1* and *Nrf2* (*Nfe2l2*) genes was monitored by qPCR as previously described [23] with slight modifications. Briefly, the qPCR reactions were performed in duplicate in a final volume of 20 µL using TB Green PreMix ex Taq II Master Mix (Takara Bio Europe, France) and 200 nM primers (Table 1), in a RotorGene 6000 instrument (Corbett life science, Sydney, Australia). The amplification conditions were 95 °C for 10 min, 40 cycles at 95 °C for 10 s and 60 °C for 50 s. To confirm the absence of non-specific products or primer dimers, a melting curve analysis was performed from 65 to 95 °C at the end of each run, with a slope of 1 °C/s, and 5 s at each temperature. A duplicate non-template control was included for each target as a negative control. *Gapdh* (glyceraldehydes-3-phosphate dehydrogenase) and *Gusb* (β-D-glucuronidase) were used as reference genes. The relative expression levels were calculated using the 2^−ΔΔCt^ method [24].

### 2.8. Thiol Content Determination by High Performance Liquid Chromatography (HPLC)

Thiol content was assayed as previously described [27]. Briefly, after treatment, cells were lysed with 100 μL of a buffer consisting of 0.1% (*v/v*) Triton X-100, 0.1 M Na_2_HPO_4_, 5 mM EDTA, pH 7.5. Fifteen μL of 0.1 N HCl and 140 μL of precipitating solution (100 mL containing 1.67 g of glacial metaphosphoric acid, 0.2 g of disodium EDTA, 30 g of NaCl) were then added. After centrifugation, the pellet was dissolved in 0.1 M NaOH and protein concentration was determined by the Bradford assay; the supernatant was mixed with 25% (*v/v*) 0.3 M Na_2_HPO_4_, and 10% (*v/v*) 5,5′-dithio-bis-(2-nitrobenzoic acid) (DTNB) was then immediately added. The mixture was stirred for 1 min at RT, then left at RT for another 5 min, and finally used for determination of GSH and other thiols by a high-performance liquid chromatography (HPLC) method. Quantitative measurements were obtained by injection of standards of known concentrations and values were normalized on protein concentration.

### 2.9. Lactate Dehydrogenase (LDH)-Based Cytotoxicity Assay

Cell growth inhibition and cell death were assessed by using a modified version of the assay described by Smith et al. [28]. Briefly, 10,000 cells/well were seeded in 96 well tissue culture plates for two days and then treated with different concentrations of I-152. Untreated cells were used as controls (CTR). Each condition was assayed in octuplicate. After 24 h and 48 h of treatment, the medium deriving from four wells for each condition was recovered and pooled in a tube; care was taken not to disturb the underlying cell layer. Two % (*v/v*) Triton X-100 was added to the remaining four wells and mixed thoroughly using a pipette to ensure complete cell lysis. The pooled media (S) and the pooled extracts (cell lysate + medium, TOT) were then centrifuged at 1000× *g* for 10 min at 4 °C and the supernatants transferred in fresh tubes. Lactate dehydrogenase (EC 1.1.1.27) activity (LDH_act_) was assayed spectrophotometrically following the protocol described in Beutler et al. [29]. Since the culture medium contained low levels of LDH, this basal activity was subtracted from the LDH_act_ determined in the samples before calculation.

Percent killing: (LDH_act_ S/LDH_act_ TOT) × 100 (1)

Percent total effect: [1-(LDH_act_ I-152 TOT/LDH_act_ CTR TOT)] × 100 (2)

Percent growth inhibition: Percent total effect (Equation (2))—percent killing (Equation (1)).

### 2.10. Statistical Analysis

Data were analyzed using Prism software version 5.0 (GraphPad, San Diego, CA, USA). The *t*-test was performed to compare two groups of data, whereas one-way ANOVA was used to compare more than two groups. The Tukey posttest was used when every mean was compared to every other mean, whereas the Dunnet posttest was used to compare every mean to the control mean. Asterisks indicate significance versus control (denoted by 0 or CTR) unless otherwise specified, and *p* ≤ 0.05 were considered significant.

## 3. Results

### 3.1. I-152 Increases the Levels of NRF2

The ability of I-152 to increase intracellular GSH levels has been previously demonstrated in vivo, in a murine model of retroviral infection [30] and in vitro, in human monocyte-derived macrophages, peritoneal murine macrophages and RAW 264.7 cells [21,27]. Since overlapping results have been obtained in primary and immortalized cells, the latter were selected as an experimental model to study how I-152 influences GSH homeostasis at the molecular level.

Previous evidence indicates that 10 mM I-152 causes GSH depletion in RAW 264.7 cells but yields a high content of thiol species in the form of NAC, MEA and I-152. By contrast, 1 mM I-152 increases cellular GSH content [27]. Thus, this range of concentrations was initially used to test whether the molecule is able to activate the NRF2 signaling pathway, considering that both MEA and NAC have been reported as potential NRF2 activators [31,32].

**Figure 1:**
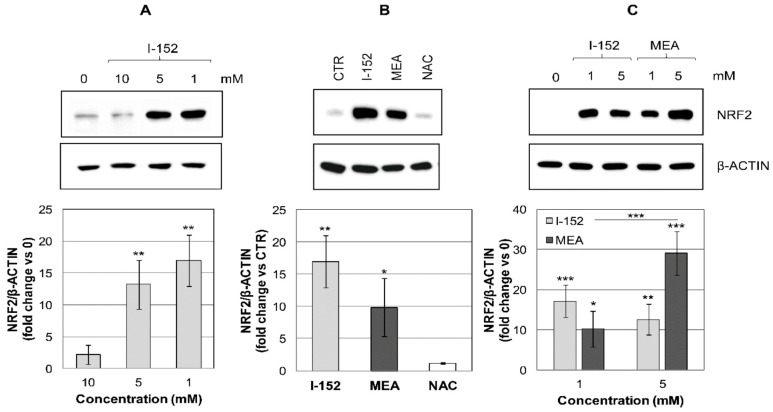
NRF2 levels in cells treated with I-152 and its metabolites NAC and MEA. RAW 264.7 cells were treated with: (**A**) different concentrations of I-152; (**B**) 1 mM I-152 compared to equimolar concentrations of NAC or MEA; (**C**) I-152 or MEA at two different concentrations. Cells were incubated with the molecules for 2 h while control cells (denoted by 0 or controls (CTR)) were incubated with fresh medium. Whole cell lysates (10 µg) were separated on 8% (*w/v*) SDS-polyacrylamide gels, blotted onto PVDF membranes and probed with an antibody against NRF2. β-ACTIN was stained as a control. Immunoreactive bands were quantified with the Image Lab software and NRF2 levels, normalized on β-ACTIN, expressed as fold-change relative to CTR (0). Values are the mean ± S.D. of at least three independent experiments. * *p* < 0.05; ** *p* < 0.01, *** *p* < 0.001.

As shown in Figure 1A, 10 mM I-152 did not affect NRF2 levels, while 5 and 1 mM significantly increased NRF2 expression.

When NAC and MEA were tested singularly, only MEA activated NRF2; by contrast, NAC had no effect (Figure 1B). Notably, MEA increased NRF2 levels dose-dependently; by contrast, the efficacy of I-152 was inversely correlated with its concentration (Figure 1C). Overall, these results suggest that I-152 may activate the NRF2 pathway, probably by delivering MEA.

**Figure 2:**
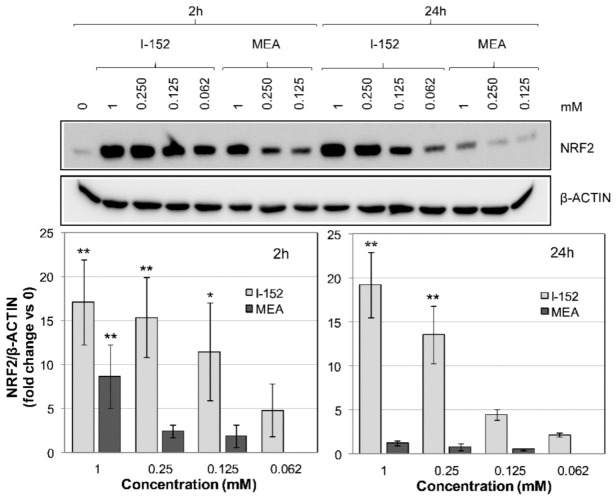
NRF2 levels in I-152- versus MEA-treated RAW 264.7 cells. Cells were treated with different concentrations of I-152 and MEA for 2 h and 24 h; control cells received only fresh medium (0). Whole cell lysates (10 µg) were separated on 8% (*w/v*) SDS-polyacrylamide gels and analyzed by immunoblotting using an antibody against NRF2. β-ACTIN was stained as a control. Immunoreactive bands were detected in a Molecular Imager and quantified with the Image Lab software. NRF2 levels, normalized to β-ACTIN content, are reported in the graph as fold change relative to control (0). Values are the mean ± SD of at least three independent experiments. * *p* < 0.05, ** *p* < 0.01.

To compare the potency of I-152 and MEA we subsequently performed dose-dependent experiments at concentrations equal to and below 1 mM. MEA is typically used in vitro at concentrations ranging from 1 mM to 0.05 mM [33,34], while doses up to 10 mM of NAC can be found in literature [35]. A stronger activation of NRF2 was observed in I-152-treated cells than in those incubated with MEA (Figure 2). Indeed, after 2 h, only 1 mM MEA significantly increased NRF2 levels over the basal level, while I-152 was effective at concentrations as low as 0.125 mM. Moreover, the effects of I-152 were still evident after 24 h incubation, while MEA was no longer effective.

### 3.2. I-152 Induces KEAP1 Oxidation and NRF2 Stabilization

**Figure 3:**
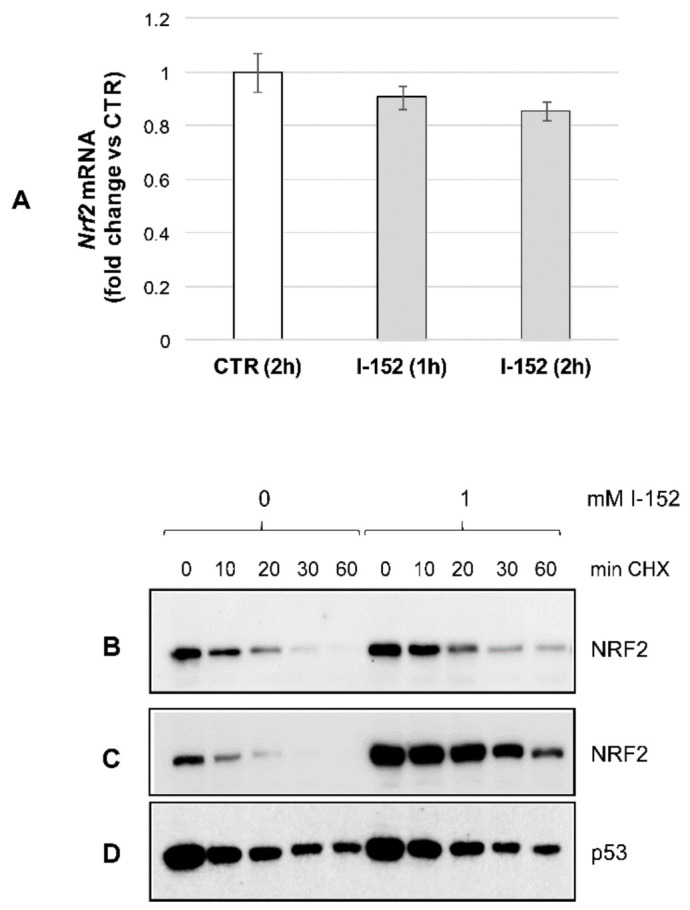
*Nrf2* mRNA levels and NRF2 protein degradation after I-152 treatment. (**A**) RTqPCR analysis of *Nrf2* mRNA levels in cells treated with 1 mM I-152 for 1 h and 2 h. Expression levels were normalized to the housekeeping genes *Gapdh* and *Gusb* and expressed as fold-change versus untreated cells (CTR). The values are the mean ± SD of two independent experiments with two technical replicates (**B**) Western immunoblotting analysis of NRF2 levels in extracts obtained from cells treated with 1 mM I-152 together with cycloheximide (CHX) for different times. Control cells were left untreated and incubated only with CHX (0). In some experiments, CHX was added after 30 min pre-incubation with I-152 and NRF2 (**C**) and p53 (**D**) protein levels were determined by western blotting. Ten µg of total proteins were loaded on 8% (*w/v*) SDS polyacrylamide gels.

To shed light on the mechanism(s) of I-152-mediated NRF2 intracellular accumulation, *Nrf2* gene expression and the rate of protein degradation were determined. After incubation with I-152, *Nrf2* mRNA levels were substantially unchanged compared to the control (Figure 3A), allowing us to exclude transcriptional induction. By contrast, co-treatment with cycloheximide, a well-known translational inhibitor, followed by Western blotting analysis revealed that NRF2 protein degradation was significantly reduced in I-152-treated cells compared to untreated cells receiving only CHX (0) (Figure 3B). More marked differences were obtained when CHX was added to the culture medium after 30 min pre-incubation with I-152 (Figure 3C).

The relative NRF2 half-life was 16.7 min and 28.4 min in I-152-treated cells depending on whether CHX was added together with I-152 or after pre-incubation with I-152, respectively versus 8.2 ± 0.47 min in untreated cells (Figure S1A,B). By contrast, degradation of p53, another short half-life protein, was unaffected, suggesting that the effect of I-152 is specific for NRF2 (Figure 3D).

**Figure 4:**
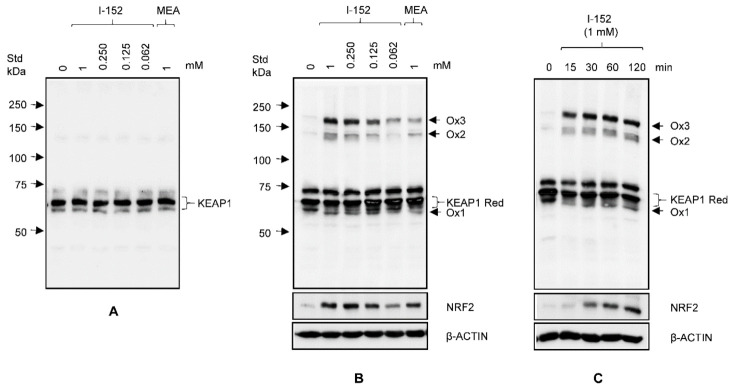
KEAP1 oxidation in cells treated with I-152 and MEA. RAW 264.7 cells were exposed to different concentrations of I-152 and to 1 mM MEA for 2 h. The cells were then lysed in the presence of NEM to block free sulfhydryl groups. The lysates were separated by reducing (**A**) and non-reducing (**B**) SDS-PAGE, and KEAP1 was revealed by western immunoblotting analysis using an anti-KEAP1 antibody. Ten µg of total proteins were loaded on 8% (*w/v*) polyacrylamide gel. (**C**) Raw 264.7 cells were treated with 1 mM I-152 for different times and lysates, obtained as in A, were separated under non-reducing conditions. In panels B and C, the blots were re-probed with anti NRF2 antibody and β-ACTIN was stained as a control. Arrows on the right side indicate the oxidized faster migrating (Ox1) and the oxidized slower migrating (Ox2 and Ox3) KEAP1 species, while brackets show the position of reduced (Red) KEAP1.

In light of this evidence, we then investigated the redox state of endogenous KEAP1 by NEM-alkylated redox western, which makes it possible to detect protein oxidation by monitoring changes in electrophoretic mobility under non-reducing conditions. After treatment, cell extracts were resolved by SDS-PAGE under non-reducing and reducing conditions and stained with an antibody against KEAP1. In all the reduced samples, KEAP1 migrated as a single band of the expected molecular weight (about 70 kDa) (Figure 4A), although a faint band of lower molecular weight could be detected in all the samples.

When reduction was omitted, KEAP1 still migrated as a major band of the same molecular mass (denoted by Red for reduced), but a second less intense band with faster mobility (denoted by Ox1 for oxidized form 1) appeared in I-152- and MEA-treated samples, but not in lysates from untreated cells (0) (Figure 4B). Moreover, a 75 kDa immunoreactive species was present in all the samples, independently of the treatment. Finally, two bands (denoted by Ox2 and Ox3) with a much slower mobility than red KEAP1 were evident in the upper part of the non-reducing gel, while they were absent in reduced samples. The intensity of the Ox1, Ox2, and Ox3 bands increased with the I-152 dose and was more pronounced in cells treated with 1 mM I-152 than in those treated with an equimolar concentration of MEA, suggesting that I-152 is a more potent KEAP1 oxidant than MEA (Figure 4B). Notably, NRF2 staining indicated that there was a good correlation between KEAP1 oxidation and NRF2 stabilization, pointing to a cause and effect relationship. The link between KEAP1 oxidation and NRF2 stabilization was further demonstrated by a time course analysis. As shown in Figure 4C, KEAP1 was already strongly oxidized after 15 min incubation with 1 mM I-152, while NRF2 levels were significantly increased over the control levels at 30 min, a time lag which is consistent with the estimated NRF2 half-life between 15 and 30 min [36,37].

### 3.3. I-152 Activates NRF2-Dependent Gene Transcription and Increases GCLM Protein Levels

**Figure 5:**
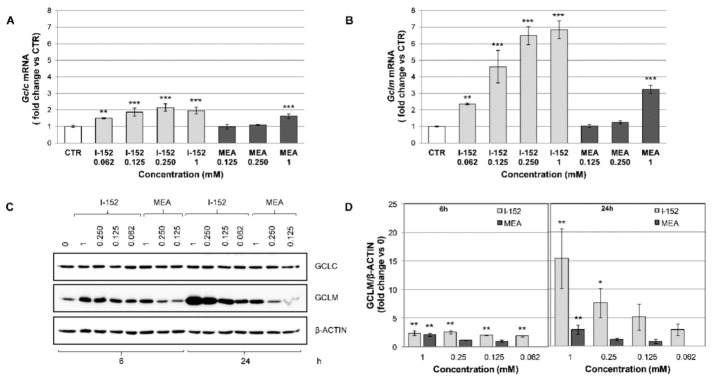
NRF2-dependent gene transcription and protein expression. RAW 264.7 cells were exposed to different concentrations of I-152 and MEA for 6 h and 24 h. (**A**,**B**) RTqPCR analysis of *Gclc* and *Gclm* mRNA levels after 6 h of treatment. mRNA levels were normalized to the housekeeping genes *Gapdh* and *Gusb* and expressed as fold-change versus untreated cells (CTR). The values are the mean ± SD of at least two independent experiments with two technical replicates. (**C**) Western immunoblotting analysis of GCLC and GCLM protein expression. Cell lysates (5 μg) were separated on 10% (*w/v*) gels and immunoblotted with specific antibodies against the target proteins. β-ACTIN was stained as a loading control. (**D**) Immunoreactive bands were quantified with the Image Lab software. GCLM levels, normalized to β-ACTIN content, are reported in the graphs as fold change relative to control (0). Values are the mean ± SD of at least three independent experiments. * *p* < 0.05; ** *p* < 0.01, *** *p* < 0.001.

To assess whether accumulation of NFR2 resulted in increased transcriptional activity, the mRNA levels of heme oxygenase-1 (*Hmox1*), a prototypical NRF2 target gene, *Gclc* and *Gclm* were determined by RTqPCR. At 6 h of treatment, all the genes were activated by all the I-152 doses tested and by 1 mM MEA (Figure S2A, Figure 5A,B). The most relevant induction was observed in the case of the *Gclm* gene (Figure 5B).

Analysis of the protein levels reveals that no changes occurred in GCLC intracellular concentrations (Figure 5C and Figure S2C), while both HMOX1 (Figure S2B) and GCLM (Figure 5C,D) protein levels were increased at 6 h incubation and rose further at 24 h in the case of GCLM. In agreement with mRNA induction, GCLM protein accumulated only in cells treated with 1 mM MEA (Figure 5C,D).

### 3.4. I-152 Increases Intracellular Thiol Content and Dose-Dependently Modulates GSH Levels

**Figure 6:**
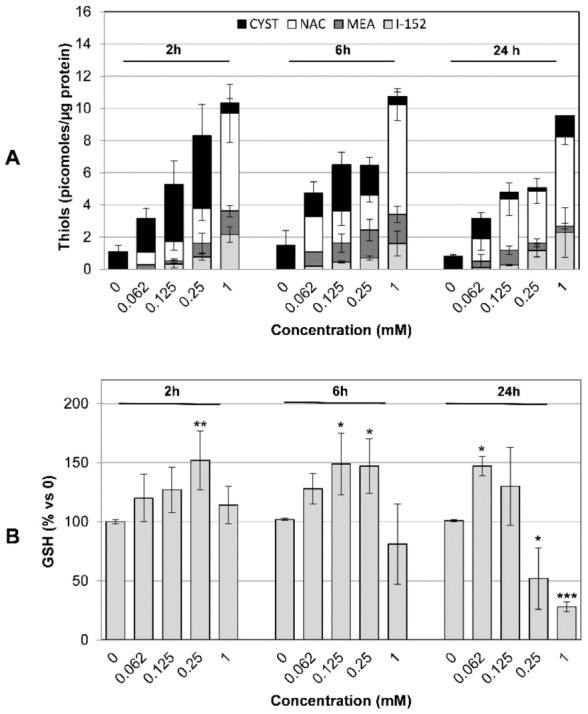
Thiol species in I-152 treated cells. MEA, NAC, I-152, cysteine (**A**) and GSH (**B**) content in RAW 264.7 cells treated with different concentrations of I-152 for 2, 6, and 24 h. After incubation, cells were washed and lysed; the lysate was then treated with precipitating solution and centrifuged. Thiol species and GSH levels were determined in the lysate supernatant by HPLC, while protein content was quantified spectrophotometrically in the lysate pellet. Quantification of thiol species was obtained by injection of standards of known concentrations and values were normalized on protein concentration. GSH content is expressed as the percent of the value obtained in untreated cells (0). Values are the mean ± SD of five independent experiments. * *p* < 0.05; ** *p* < 0.01, *** *p* < 0.001.

At 2 h incubation, analysis of the free thiol pools showed that cysteine represented the most abundant thiol species, GSH excluded, except in cells treated with 1 mM I-152, where NAC was predominant (Figure 6A). Notably, at 24 h incubation, a predominance of NAC was observed at all the I-152 doses tested. Although I-152 is expected to liberate equimolar concentrations of its metabolites, intracellular MEA levels were markedly lower than those of NAC, suggesting that MEA could be partially converted into cystamine upon oxidation of its sulfhydryl group and/or form mixed disulfides. Such disulfides could not be revealed by the chromatographic analysis used here since it was specific for the identification of –SH carrying molecules. Within the time frame 2–24 h, GSH content dose-dependently increased over the physiological level in cells treated with I-152 (Figure 6B). However, significant depletion of intracellular GSH was observed at 24 h, but only at the highest doses tested (i.e., 0.25 and 1 mM). Thus, I-152 dose-dependently increased intracellular thiol levels: (i) By delivering I-152, NAC and MEA; (ii) by providing high levels of cysteine and (iii) by elevating GSH content.

Increased cysteine levels together with MEA were found within the cells upon MEA administration (Figure S3A). Cysteine content was also elevated in NAC-treated cells, but NAC was detectable only when delivered at the highest dose and/or for longer times (Figure S4A). Despite higher cysteine availability, both MEA and NAC only modestly affected GSH cellular content (Figures S3B and S4B).

### 3.5. I-152 Activates the ATF4 Signaling Pathway

**Figure 7:**
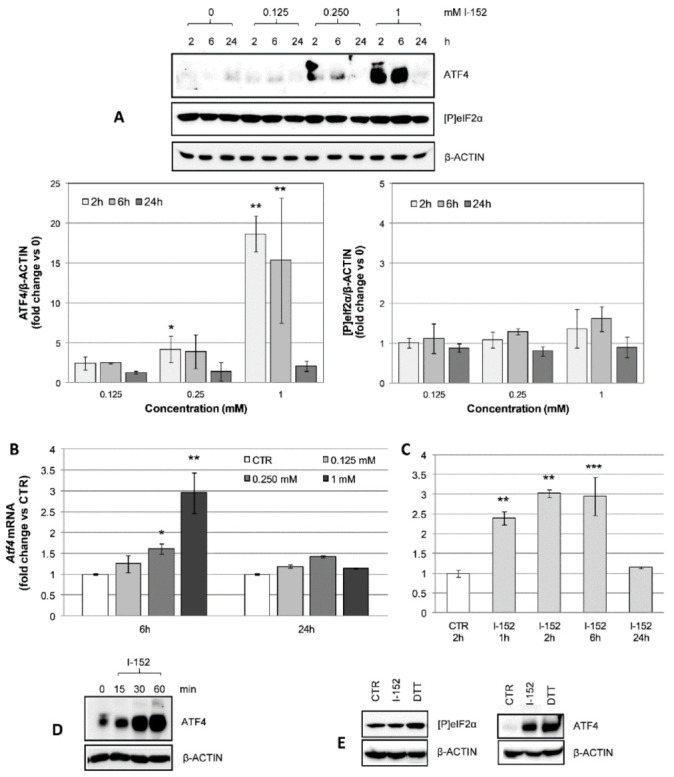
Activating transcription factor 4 (ATF4) transcriptional activation and protein accumulation in I-152-treated cells. (**A**) Cells were incubated with different concentrations of I-152 for 2 h, 6 h, and 24 h and whole lysates corresponding to 10 μg of proteins were separated on 10% (*w/v*) polyacrylamide gels. The blots were probed with anti ATF4 and anti [P]eIF2α antibodies. β-ACTIN was stained as a control. Untreated cells receiving only fresh medium served as a control (denoted by 0). Immunoreactive bands were quantified with the Image Lab software. ATF4 and [P]eIF2α levels, normalized to β-ACTIN content, are reported in the graphs as fold change relative to control (0). Values are the mean ± SD of at least three independent experiments. * *p* < 0.05, ** *p* < 0.01. (**B**) RTqPCR analysis of *Atf4* mRNA levels in cells treated with different concentrations of I-152 for 6 h and 24 h. (**C**) RTqPCR analysis of *Atf4* mRNA levels in cells treated with 1 mM I-152 for different times. mRNA levels were normalized to the housekeeping genes *Gapdh* and *Gusb* and expressed as fold-change versus untreated cells (CTR). The values are the mean ± SD of at least two independent experiments with two technical replicates. * *p* < 0.05, ** *p* < 0.01, *** *p* < 0.001. (**D**) Cells were incubated with 1 mM I-152 for different times and lysates were resolved by SDS-PAGE, immunoblotted and stained with anti ATF4. (**E**) Western immunoblotting analysis of cell extracts obtained from CTR cells and cells treated with 1 mM I-152 and 1 mM DTT for 30 min (left panel) and 1 h (right panel). ATF4 and [P]eIF2α were detected using specific antibodies, while β-ACTIN was stained as a loading control.

Like any other biological molecule, GSH levels largely depend on its rate of synthesis and degradation. Thus, we reasoned that at the highest I-152 concentrations tested, GSH overproduction might lead to reductive stress and result in the activation of degradative pathways to rebalance redox homeostasis [38]. GSH depletion has often been associated with ATF4 activation and CHAC1 expression, the latter of which controls intracellular GSH degradation under stressful conditions. ATF4 was indeed strongly activated by 1 mM I-152 and modestly activated by 0.25 mM via a signaling pathway not dependent on eIF2α phosphorylation, which was unaffected by the treatment (Figure 7A), but dependent on *Atf4* transcriptional induction (Figure 7B).

At 6 h, both the mRNA and protein levels were still high to return to basal levels at 24 h (Figure 7A,B). Time course analysis of *Atf4* mRNA expression revealed that at 1 h, the transcription was fully activated (Figure 7C). Subsequent analysis of the kinetics of protein accumulation demonstrated that ATF4 protein levels started to increase as soon as 15 min after I-152 treatment (Figure 7D), highlighting an unexpectedly fast activation of the ATF4 signaling. To further exclude the involvement of the ISR pathway, cells were treated in parallel with I-152 and dithiotreitol (DTT), which is known to induce ER stress, leading to eIF2α phosphorylation and sustained ATF4 translation [39]. As expected, levels of [P]eIF2α were increased after exposure to DTT, but not to I-152, although ATF4 accumulation was observed in both cases (Figure 7E). This clearly demonstrates that the two thiol species induce distinct signaling cascades to activate ATF4.

**Figure 8:**
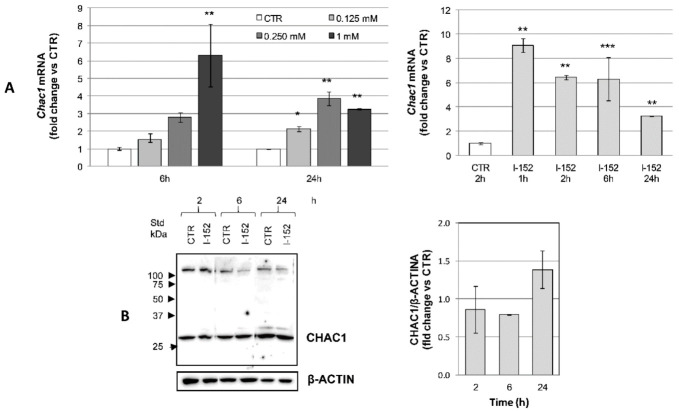
CHAC1 transcriptional activation and protein levels in I-152-treated cells. (**A**) RTqPCR analysis of *Chac1* mRNA levels in cells treated with different concentrations of I-152 for 6 h and 24 h and in cells treated with 1 mM I-152 for different times. mRNA levels were normalized to the housekeeping genes *Gapdh* and *Gusb* and expressed as fold-change versus untreated cells (CTR). The values are the mean ± SD of two independent experiments with two technical replicates (**B**) Cells were incubated with different concentrations of I-152 for 2 h, 6 h and 24 h, and whole lysates corresponding to 10 μg of proteins were separated on 12% (*w/v*) polyacrylamide gels. The blots were probed with anti CHAC1 antibody. β-ACTIN was stained as a control. Untreated cells receiving only fresh medium served as a control (CTR). Immunoreactive bands were quantified with the Image Lab software. CHAC1 levels, normalized to β-ACTIN content, are reported in the graph as fold change relative to CTR. Values are the mean ± SD of at least three independent experiments. * *p* < 0.05, ** *p* < 0.01, *** *p* < 0.001.

*Chac1* mRNA induction followed the same trend reported for *Atf4* (Figure 8A), but no changes in protein expression could be observed using two different antibodies (one from GeneTex, as reported in Material and Methods, and one from Biorbyt orb100972) specifically recognizing a band of the expected molecular weight (Figure 8B and data not shown).

**Figure 9:**
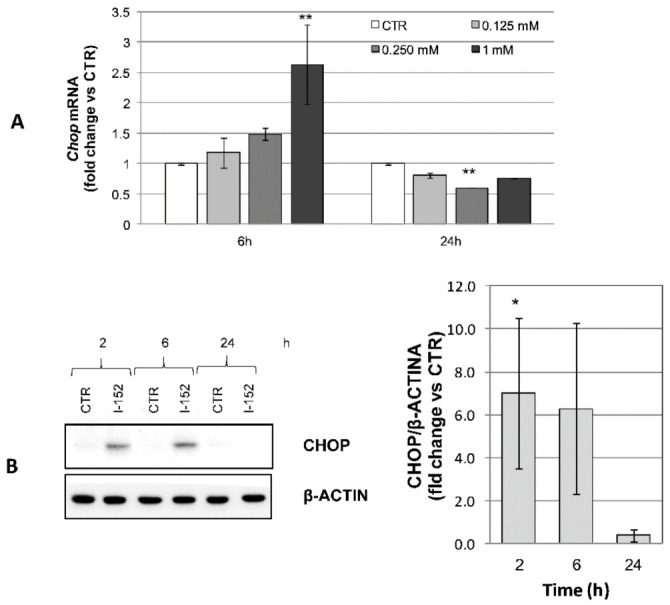
CHOP transcriptional activation and protein expression in I-152-treated cells. (**A**) RTqPCR analysis of *Chop* mRNA levels in cells treated with different concentrations of I-152 for 6 h and 24 h. mRNA levels were normalized to the housekeeping genes *Gapdh* and *Gusb* and expressed as fold-change versus untreated cells (CTR). The values are the mean ± SD of two independent experiments with two technical replicates (**B**) Cells were incubated with 1 mM I-152 for 2 h, 6 h, and 24 h and whole lysates corresponding to 20 μg of proteins were separated on 12% (*w/v*) polyacrylamide gels. The blots were probed with anti CHOP antibody. β-ACTIN was stained as a control. Untreated cells receiving only fresh medium served as a control (CTR). Immunoreactive bands were quantified with the Image Lab software. GHOP levels, normalized to β-ACTIN content, are reported in the graphs as fold change relative to CTR. Values are the mean ± SD of at least three independent experiments. * *p* < 0.05; ** *p* < 0.01.

By contrast, the ATF4 gene target *GADD153* (*Chop*) was induced both at the transcriptional and protein level consistently with ATF4 activation, indicating that ATF4 was transcriptionally competent (Figure 9A,B).

**Figure 10:**
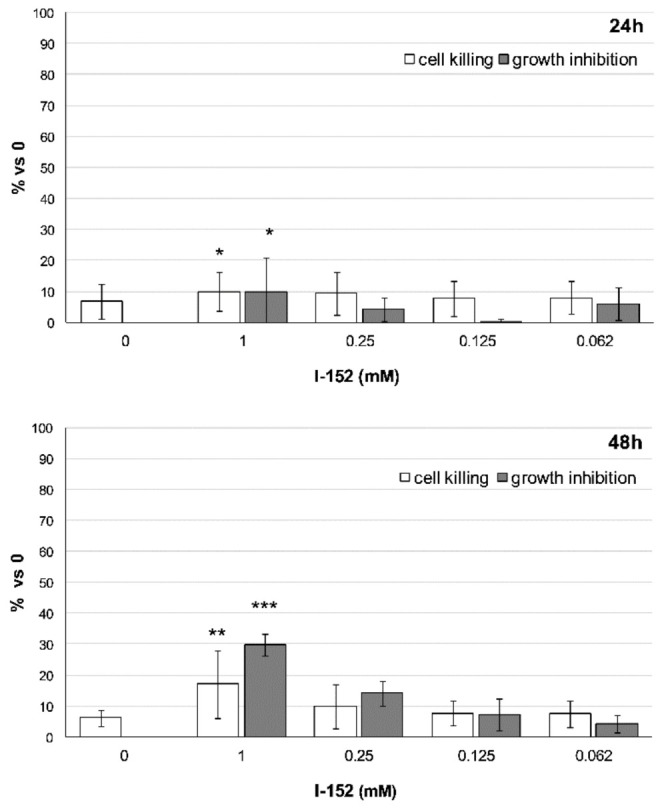
Cell killing and growth inhibition after I-152 treatment. Cells were incubated with different doses of I-152 for 24 h and 48 h. Untreated cells receiving only fresh medium served as a control (denoted by 0). LDH activity was measured for each condition in the cell culture medium and, in parallel, in the medium after complete cell lysis with Triton-X100. This method makes it possible to distinguish cell death versus growth inhibition as described in materials and methods. Data are reported in the graph as fold change relative to control (0). The values are the mean ± SD of two independent experiments with four technical replicates. * *p* < 0.05, ** *p* < 0.01, *** *p* < 0.001.

Since CHOP is a well-known mediator of apoptosis, LDH-based cytotoxicity assays were performed at 24 h and 48 h of incubation with the molecule. The use of the classical MTS assay was avoided because I-152 per se induced a strong reduction of the MTS tetrazolium compound. As shown in Figure 10, only 1 mM I-152 promoted a block in cell proliferation and caused cell death. The effects were very mild at 24 h, with only 10% of the cells being affected. By contrast, these effects became more evident at 48 h, although more cells appeared to be inhibited in their growth rather than killed by the molecule.

## 4. Discussion

I-152 increases physiological GSH levels and replenishes intracellular GSH content under conditions of depletion; however, the molecular mechanisms underlying its pro-GSH activity have only been partially elucidated.

Data reported in this paper demonstrate that I-152 is a potent pro-GSH molecule since it can stimulate GSH biosynthesis by forcing GSH production through the combined delivery of NAC, which provides the rate limiting building block cysteine, and MEA, which induces NRF2 activation and GCLM protein expression. It has been shown that GCLM levels are limiting within cells, thus upregulation of GCLM alone is sufficient to increase GCL catalytic activity by increasing holoenzyme formation. The heterodimeric enzyme is more efficient in catalysing the reaction and less sensitive to feedback inhibition by GSH [2,40].

The potency of I-152 in terms of its ability to increase GSH content was far superior than that of NAC or MEA administered alone. This difference in potency can be accounted for by the different uptake mechanisms of the molecules together with the inability or low capacity of NAC and MEA, respectively, to activate the NRF2 pathway. When used alone, NAC was available early intracellularly only when used at the highest concentration tested and for long periods of incubation. This is because NAC enters by passive diffusion, but its permeability is low since it is mostly negatively charged at physiological pH. Thus, high doses and longer treatments are required to favor its cell penetration [41]. Conversely, high cysteine levels were detected in NAC-treated cells, supporting the notion that NAC may also reduce extracellular cystine to cysteine, which then enters the cells [42]. Compared to the administration of NAC alone, I-152-treated cells contained a significantly higher amount of NAC, which was also detectable at the lower doses, suggesting that I-152 enters as such and it is mostly metabolized intracellularly. Unlike NAC, MEA was readily detectable at all the doses and times tested, together with high cysteine levels. MEA uptake is thought to occur very quickly through a mechanism involving thiol-disulphide interchange with extracellular cystine to form cysteamine–cysteine mixed disulphides that enter cells through amino acid transporters and are then reduced back to cysteamine and cysteine [43]. The ability of MEA to increase GSH levels has been attributed to its capacity to release cysteine from cystine pools, but other molecular mechanisms might be involved [34]. Calkins et al. [31] provided evidence that MEA is a weak activator of the NRF2 pathway in neuronal cells. In this system, however, the oxidized form of MEA, cystamine (here named MEA_S-S_ for simplicity), displayed a much higher potency, suggesting that MEA_S-S_ rather than MEA activates NRF2. In circulation and within cells, MEA is partially converted into MEA_S-S_ after the oxidation of its sulfhydryl group. It has been widely documented that both MEA and MEA_S-S_ can affect the activity of different proteins, including transglutaminase, whose mechanism(s) of inactivation has been thoroughly studied. In particular, MEA_S-S_ but not MEA, has been shown to inhibit transglutaminase activity by an oxidative mechanism where MEA_S-S_ promotes the formation of a physiologically relevant disulfide bond between Cys370 and Cys371 [44]. A similar mechanism of disulfide interchange may be responsible for the formation of the three oxidized KEAP1 species (Ox1, 2 and 3) in cells treated with I-152. Using KEAP1 mutants, Fourquet et al. [45] demonstrated that in cells exposed to oxidants, KEAP1 is oxidized generating three byproducts: One oxidized species, corresponding to Ox1, which carries an intramolecular Cys226–Cys613 disulfide, and two high molecular weight oxidized species, corresponding to Ox2 and Ox3, involving Cys151-Cys151 mixed disulfide between two KEAP1 molecules, and KEAP1 and another polypeptide. While intramolecular disulfide formation was not essential to NRF2 activation, Cys151-Cys151 intermolecular disulfide was critical to relieving KEAP1-mediated NRF2 degradation [45]. Different conclusions were drawn when two NRF2 inducers, namely sulphoraphane and trinitrobenzene sulphonate, were tested and found to promote intra- and intermolecular disulphides, respectively [46], suggesting that KEAP-1 function might be affected by both types of modifications. It is worth noting that the above-mentioned studies were performed in cells ectopically expressing KEAP1; by contrast, our observations concern the endogenously expressed protein, highlighting the biological relevance of these oxidizing mechanisms.

I-152 is expected to release equimolar amounts of NAC and MEA; however, analysis of the thiol species revealed that MEA levels were markedly lower than those of NAC. Based on this observation, it could be speculated that the MEA moiety, released after I-152 metabolization, may be partly oxidized into MEA_S-S_, although we cannot exclude conjugation to other thiol species. Moreover, since cells also contain a certain amount of non-hydrolyzed I-152, a direct/indirect role for the I-152 molecule in NRF2 activation cannot be excluded.

The intracellular delivery of high amounts of thiol species as well as the overproduction of GSH may induce reductive stress, which, in turn, may enhance mitochondrial oxidation with the production of ROS [47]. Thus, I-152 and/or its metabolites could act as antioxidants or pro-oxidants depending on the dose used: At low doses, I-152 stimulates the expression of antioxidant enzymes, while at high doses it leads to GSH depletion and reduced cell proliferation and death. It is worth noting that NAC is also known to behave differently according to its concentration and the redox status of the cells in which it is used [48,49].

Interestingly, it has recently been shown that NAC displays powerful mitochondrial and antioxidant effects, not only as a glutathione precursor, but also by triggering mitohormesis [50]. Mitohormesis assumes that a moderate increase in ROS production during mitochondrial activity leads to the activation of cellular defense systems, leading to a long-term increase in the levels of mitochondria and antioxidant enzymes. Thus, future studies might investigate these mechanisms in cells treated with I-152. On the other hand, an excessive production of ROS could deplete GSH levels, thus damaging cells. Interestingly, at I-152 concentrations promoting GSH depletion, NRF2 activation was paralleled or even preceded by transcriptional induction of the bZIP factor ATF4. Similar results were obtained by Mimura et al. [11], who demonstrated that low-dose carnosinic acid (CA) activates the NRF2 pathway only, exhibiting moderate anti-oxidative effects; by contrast, high-dose CA activates both NRF2 and ATF4 to potentiate the NRF2 antioxidative pathway. Cooperation between NRF2 and ATF4 in modulating antioxidant gene expression has also been shown to occur in response to treatment with fisetin, a flavonoid able to stimulate GSH production [10]. In the case of fisetin, no mechanistic explanation for ATF4 induction was provided, whereas for CA, activation of the ISR pathway was shown. Moreover, GSH levels were increased by low doses of CA, whereas they were unchanged by high dose administration, despite induction of GCLM and GCLC mRNA levels. Since the authors found induction of CHAC1 mRNA expression, they speculated that higher concentrations of CA could deplete GSH by ATF4-mediated induction of CHAC1 to reestablish homeostasis.

In our experimental system, I-152 did not induce ISR activation, as demonstrated by the fact that eIF2α phosphorylation was unaffected by the treatment. Moreover, despite strong induction of CHAC1 mRNA levels, protein levels were unchanged, allowing us to exclude the activation of compensatory degradative GSH pathways. At present, the reason why CHAC1 mRNA induction does not result in higher protein expression is unknown. Difficulty in detecting the CHAC1 protein despite high mRNA expression has been reported [51]. On the contrary, ATF activation was accompanied by the expression of its target gene CHOP, as well as by the inhibition of cell proliferation together with a small percentage of dead cells observed at 24 h and 48 h treatment with 1 mM I-152. ATF4 controls life-death decisions after stress, switching from adaptive to pro-apoptotic gene expression [52]. This switch has been attributed in part to the formation of different ATF4 heterodimers that control the expression of specific sets of gene targets. It has been proposed that dimerization with its target gene CHOP signals apoptosis [53,54], although ATF4 and CHOP may cooperate to induce genes mediating stress relief and cell survival such as genes encoding for proteins involved in autophagy [55]. Thus, further experiments will aim to assess the consequence of ATF4/CHOP co-activation in terms of pro-survival/pro-apoptotic gene expression. Another aspect that warrants investigation is the mechanism underlying transcriptional induction of ATF4. ATF4 gene transcription is induced in response to different stresses and is mediated by different transcription factors, including NRF2 and ATF4 itself [56,57]. Since NRF2 was activated by all the I-152 doses tested, while ATF4 was induced only at the highest concentrations, its involvement appears unlikely. Moreover, the increase in ATF4 protein levels preceded NRF2 protein accumulation in time course experiments again allowing us to exclude a cause and effect relationship between NRF2 activation and ATF4 transcriptional induction. Finally, the half-life of ATF4 was unchanged in the presence of I-152 (unpublished data), thus excluding the possibility that the factor, which is intrinsically unstable, could become stabilized by I-152 treatment, activating its own expression.

## 5. Conclusions

In conclusion, the data presented herein provide evidence that I-152 efficiently delivers NAC and MEA within the cells, exerting unique effects which are also dependent on the dose applied. At high and low doses (0.062-1 mM), KEAP1 is oxidized and NRF2 is stabilized, leading to transcriptional induction of its target genes, including GCLM. GCLM overexpression together with co-delivery of cysteine precursors boosts GSH production. At high I-152 doses (0.25–1 mM), however, GSH levels tend to decrease over time, leading to reduced proliferation and ultimately resulting in cell death (1 mM I-152). Interestingly, this effect was accompanied by an early activation of ATF4 and ATF4-dependent gene transcription (i.e., CHOP and CHAC1), as part of a stress response which is not mediated by the classical ISR pathway. Although NRF2 and ATF4 have been reported to concur in enhancing GSH levels and mounting the antioxidant response, in this context, the co-activation of ATF4 is probably necessary to switch from the adaptive response to pro-apoptotic signaling. ATF4 has been described as a redox-regulated pro-death transcription factor in neuronal cells where it seems to positively influence ROS levels and increase glutathione consumption [58].

I-152 represents the very first attempt to combine two pro-GSH molecules to improve their potency. The co-drug approach, which involves linking two drugs via a cleavable covalent bond, represents a novel strategy for enhancing GSH levels [59]. The approach makes it possible to improve membrane permeability and antioxidant activity compared to parent compounds [60]. Moreover, because of the potential synergism and cross-reactivity between the released compounds, the co-delivery of different thiol species may lead to unpredictable outcomes warranting further investigation.

## Figures and Tables

**Table 1:** Primers used for gene expression analysis in RAW 264.7 cells.

Target mRNA	Accession Number	Forward Primer (5′–3′)	Reverse Primer (5′–3′)
*Atf4*	NM_001287180	GCAGTGTTGCTGTAACGGAC	ATCTCGGTCATGTTGTGGGG
*Chac1*	NM_026929	TATAGTGACAGCCGTGTGGG	GCTCCCCTCGAACTTGGTAT
*Chop*	NM_007837	GAGTCCCTGCCTTTCACCTT	TTCCTCTTCGTTTCCTGGGG
*Gclc*	NM_010295	GGAGAGGACAAACCCCAACC	CTCAGACATCGTTCCTCCGT
*Gclm*	NM_008129	GGAACCTGCTCAACTGGGG	GGTCTTTTGGATACAGTCCCGA
*Hmox1*	NM_010442	TTAAGCTGGTGATGGCTTCCT	AGTGGGGCATAGACTGGGTT
*Nrf2*	NM_010902	CACATTCCCAAACAAGATGCCT	TATCCAGGGCAAGCGACTCA
*Gusb*	NM_010368	GGGTGTGGTATGAACGGGAA	CCATTCACCCACACAACTGC
*Gapdh*	NM_001289726	TGCCCCCATGTTTGTGATG	TGTGGTCATGAGCCCTTCC

*Atf4*, Mus musculus activating transcription factor 4; *Chac1*, Mus musculus ChaC, cation transport regulator 1; *Chop*, Mus musculus DNA-damage inducible transcript 3; *Gclc*, Mus musculus glutamate-cysteine ligase, catalytic subunit; *Gclm*, Mus musculus glutamate-cysteine ligase, modifier subunit; *Hmox1*, Mus musculus heme oxygenase 1; *Nrf2*, Mus musculus nuclear factor, erythroid derived 2, like 2; *Gapdh*, Mus musculus glyceraldehyde-3-phosphate dehydrogenase; *Gusb*, Mus musculus β-D-glucuronidase. *Atf4*, *Chac1* and *Chop* primers were described in [23]; *Nrf2* primers were taken from [25] and *Gapdh* primers from [26].

## Data Availability

The data presented in this study are available within the article and in supplementary material.

## Supplementary Materials

The following are available online at https://www.mdpi.com/2076-3921/10/2/175/s1, Figure S1: NRF2 relative half-life, Figure S2: HMOX1 and GCLC expression, Figure S3: NAC, cysteine and GSH content in NAC-treated cells, Figure S4: MEA, cysteine and GSH content in MEA-treated cells.

## Abbreviations

ATF4	activating transcription factor 4
CA	carnosinic acid
CHAC1	cation transport regulator-like protein 1
CHOP	C/EBP-homologous protein (CHOP10/GADD153)
CHX	cycloheximide
DTT	dithiothreitol
eIF2α	eukaryotic translation initiation factor 2α
GAPDH	glyceraldehyde-3-phosphate dehydrogenase
GCLC	ɣ-glutamyl-cysteine ligase catalytic subunit
GCLM	ɣ-glutamyl-cysteine ligase modifier subunit
GSH	glutathione
GUSB	β-D-glucuronidase
HMOX1	heme oxygenase 1
HPLC	high performance liquid chromatography
IRS	integrated stress response
KEAP1	kelch-like ECH-associated protein 1
LDH	lactate dehydrogenase
MEA	cysteamine/β-mercaptoethylamine
MEAs-s	cystamine
NAC	*N*-acetyl-cysteine
NEM	*N*-ethylmaleimide
NRF2	nuclear factor E2-related factor 2
SMEA	S-acetyl-cysteamine
