# Supplementary material for: Activation of NRF2 and ATF4 Signaling by the Pro-Glutathione Molecule I-152, a Co-Drug of N-Acetyl-Cysteine and Cysteamine
Source: Antioxidants (Basel). 2021 Jan 26;10(2):175. doi: 10.3390/antiox10020175 (PMC7911873; doi:10.3390/antiox10020175)
Supplement: Supplementary file 1 [file antioxidants-10-00175-s001.pdf]

SUPPLEMENTARY FIGURES

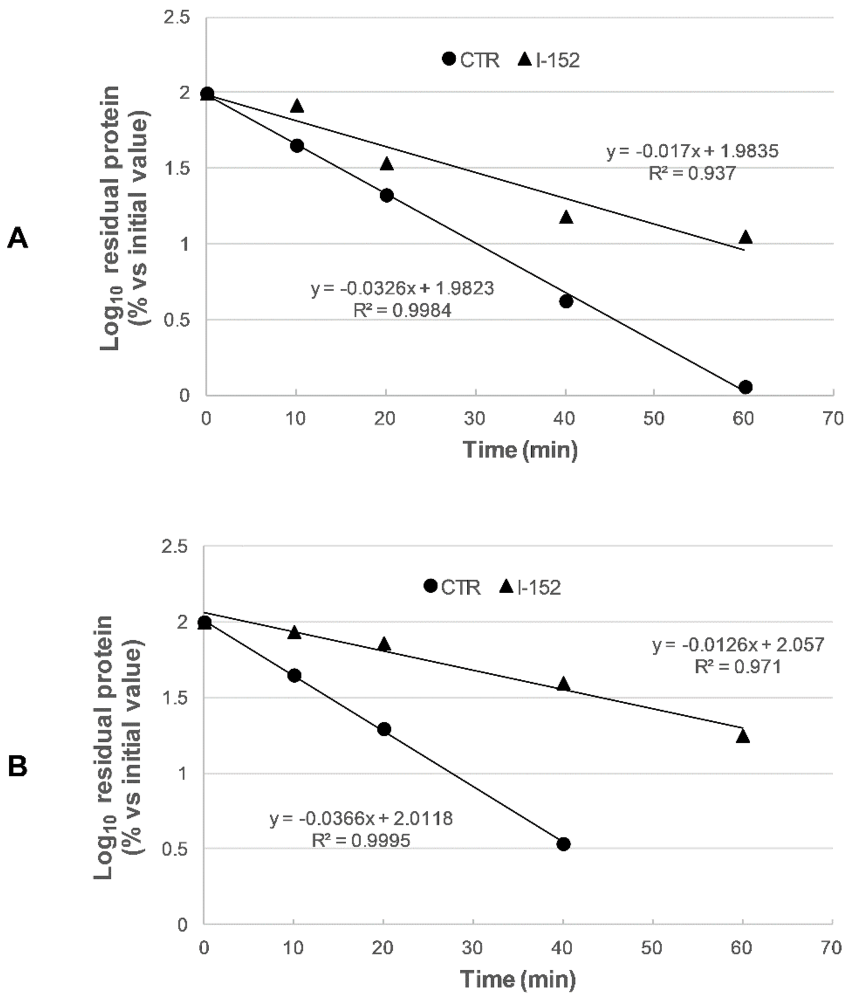

Figure S1. *NRF2 relative half-life***.** Intensities of the immunoreactive bands shown in Figure 3B (**A**) and 3C (**B**) were quantified and expressed as the percent of the band intensity of the corresponding time 0 sample (initial value) to obtain the percent of the residual protein at each time point. The Log10 of percent values were plotted as a function of time and linear regression analysis was carried out on the data to determine NRF2 relative half-life as described in Material and Methods.

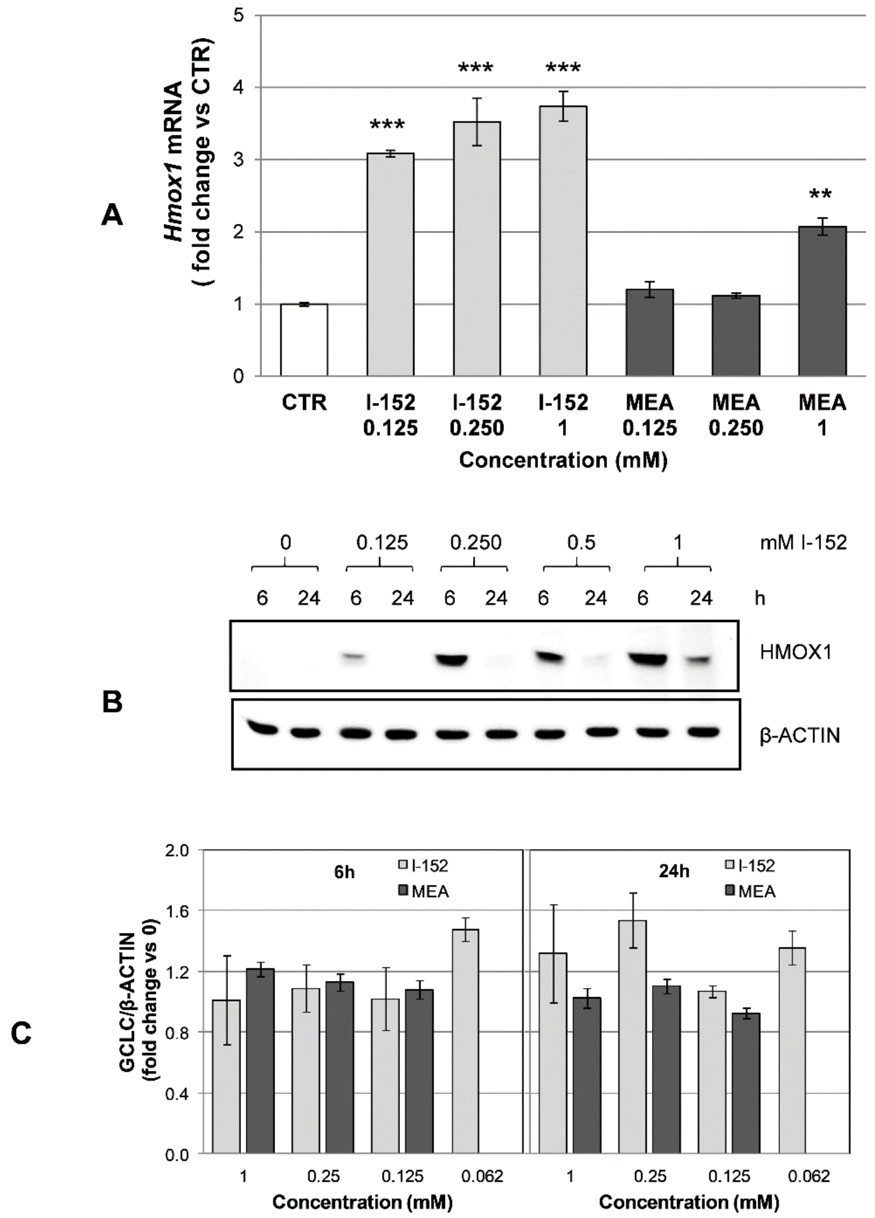

Figure S2. *HMOX1 and GCLC expression.* **(A)** RTqPCR analysis of *Hmox1* mRNA levels after 6h of treatment. mRNA levels were normalized to the housekeeping genes *Gapdh* and *Gusb* and expressed as fold-change versus untreated cells (0). The values are the mean ± SD of two independent experiments with two technical replicates **(B)** RAW 264.7 cells were exposed to different concentrations of I-152 and MEA for 6h and 24h. Cell lysates (10 μg) were separated on 10% (w/v) gels and immunoblotted with a specific antibody. β-ACTIN was stained as a loading control. **(C)** Quantification of GCLC protein levels. Immunoreactive bands were quantified with the Image Lab software. GCLC levels, normalized to β-ACTIN content, are reported in the graphs as fold change relative to control (0). Values are the mean ± SD of at least three independent experiments. **p<0.01, ***p<0.001.

A

B

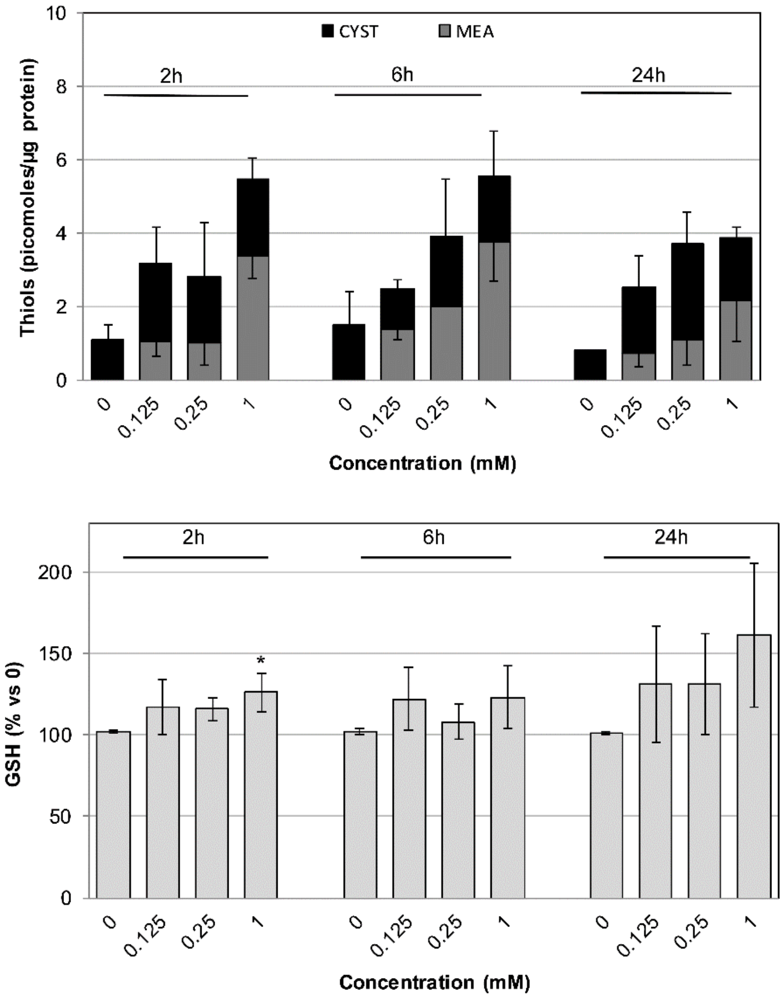

Figure S3. *NAC, cysteine and GSH content in NAC-treated cells.* NAC, cysteine **(A)** and GSH content **(B)** in RAW 264.7 cells treated with different concentrations of NAC for 2, 6, and 24h. After incubation, cells were washed and lysed; the lysate was then treated with precipitating solution and centrifuged. Thiol species and GSH levels were determined in the lysate supernatant by HPLC, while protein content was quantified spectrophotometrically in the lysate pellet. Quantification of thiol species was obtained by injection of standards of known concentration and values were normalized on protein concentration. GSH content is expressed as the percent of the value obtained in untreated cells (0). Values are the mean ± SD of five independent experiments. *$p < 0.05$ vs untreated control (0).

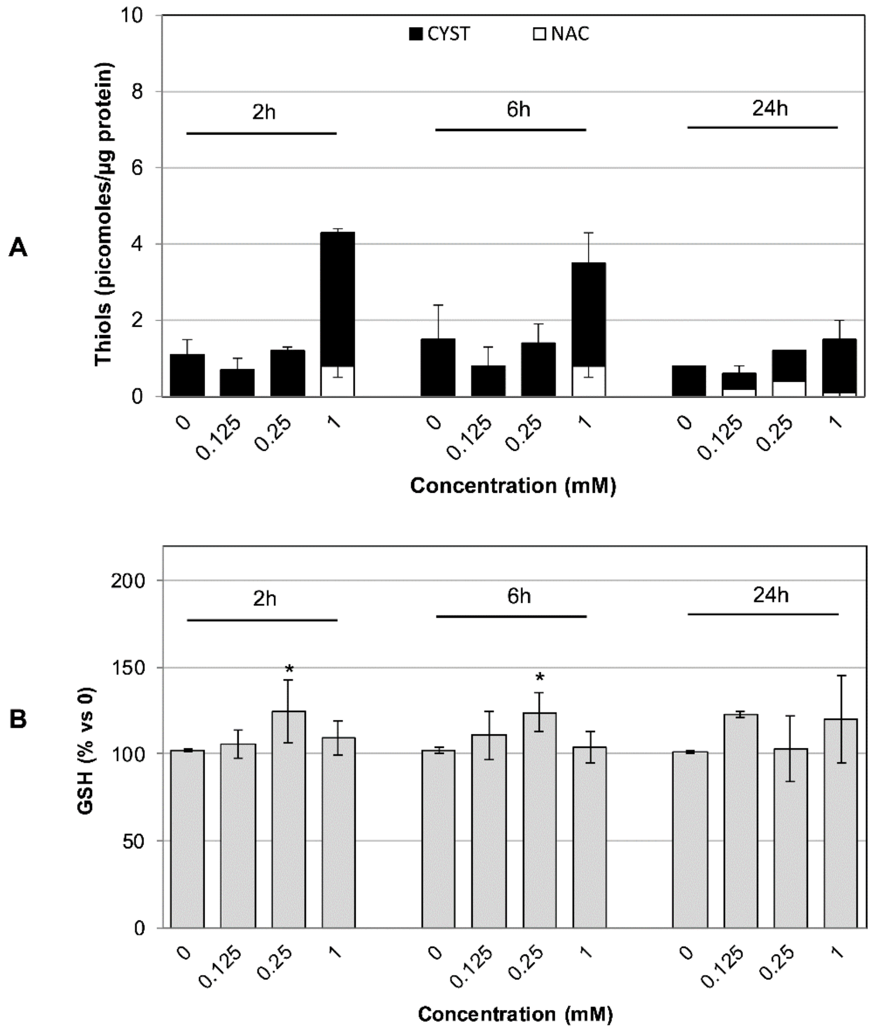

Figure S4. *MEA, cysteine and GSH content in MEA-treated cells.* MEA, cysteine **(A)** and GSH content **(B)** in RAW 264.7 cells treated with different concentrations of MEA for 2, 6, and 24h. After incubation, cells were washed and lysed; the lysate was then treated with precipitating solution and centrifuged. Thiol species and GSH levels were determined in the lysate supernatant by HPLC, while protein content was quantified spectrophotometrically in the lysate pellet. Quantification of thiol species was obtained by injection of standards of known concentration and values were normalized on protein concentration. GSH content is expressed as the percent of the value obtained in untreated cells (0). Values are the mean ± SD of five independent experiments. *$p < 0.05$ vs untreated control (0).
